# Disseminated intravascular coagulation: new identity as endotheliopathy-associated vascular microthrombotic disease based on in vivo hemostasis and endothelial molecular pathogenesis

**DOI:** 10.1186/s12959-020-00231-0

**Published:** 2020-10-14

**Authors:** Jae C. Chang

**Affiliations:** Department of Medicine, University of California School of Medicine, Irvine, CA 92603 USA

**Keywords:** Disseminated intravascular coagulation, Disseminated intravascular microthrombosis, Endotheliopathy, Hemostasis, Thrombosis, Fibrinogenesis, Fibrin clots, Hepatic coagulopathy, Macrothrombogenesis, Microthrombogenesis, Thrombotic thrombocytopenic purpura, TTP-like syndrome, Vascular microthrombotic disease

## Abstract

Disseminated intravascular coagulation (DIC) can be correctly redefined as disseminated intravascular microthrombosis based on “two-path unifying theory” of in vivo hemostasis. “DIC” is a form of vascular microthrombotic disease characterized by “microthrombi” composed of platelets and unusually large von Willebrand factor multimers (ULVWF). Microthrombotic disease includes not only “DIC”, but also microthrombosis occurring in thrombotic thrombocytopenic purpura (TTP), TTP-like syndrome, and focal, multifocal and localized microthrombosis. Being a hemostatic disease, microthrombotic disease occurs as a result of lone activation of ULVWF path via partial in vivo hemostasis. In endothelial injury associated with critical illnesses such as sepsis, the vascular damage is limited to the endothelial cell and activates ULVWF path. In contrast, in intravascular traumatic injury, the local damage may extend from the endothelial cell to subendothelial tissue and sometimes beyond, and activates both ULVWF and tissue factor (TF) paths. When endotheliopathy triggers exocytosis of ULVWF and recruits platelets, ULVWF path is activated and promotes microthrombogenesis to produce microthrombi composed of microthrombi strings, but when localized vascular damage causes endothelial and subendothelial tissue damage, both ULVWF and TF paths are activated and promote macrothrombogenesis to produce macrothrombus made of complete “blood clots”. Currently, “DIC” concept is ascribed to activated TF path leading to fibrin clots. Instead, it should be correctly redefined as microthrombosis caused by activation of ULVWF path, leading to endotheliopathy-associated microthrombosis. The correct term for acute “DIC” is disseminated microthrombosis-associated hepatic coagulopathy, and that for chronic “DIC” is disseminated microthrombosis without hepatic coagulopathy. TTP-like syndrome is hematologic phenotype of endotheliopathy-associated microthrombosis. This correct concept of “DIC” is identified from novel theory of “*in vivo* hemostasis”, which now can solve every mystery associated with “DIC” and other associated thrombotic disorders. Thus, sepsis-associated coagulopathy is not “DIC”, but is endotheliopathy-associated vascular microthrombotic disease.

## Background

Since the introduction of term disseminated intravascular coagulation (DIC) by Donald McKay [1] early 1950s for a mysterious thrombo-hemorrhagic syndrome, many blood coagulation scientists, pathologists and clinicians have ardently searched for the genuine nature of this most feared and deadly disease more than 60 years. Past several decades, hundreds of articles have been published in pursuit of its pathogenesis alone on DIC. However, the true identity of DIC occurring in many critical illnesses has been remained in secrecy.

**Fig. 1:**
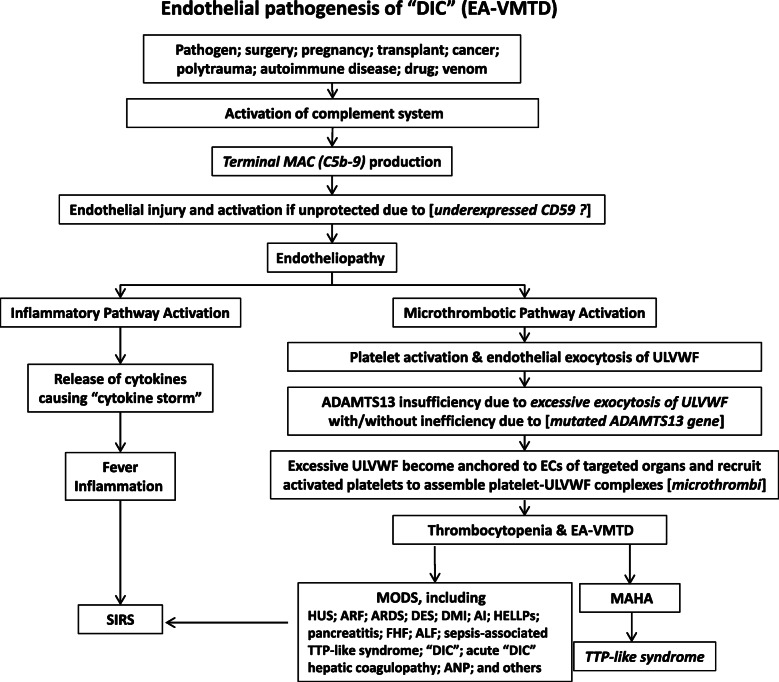
Endothelial molecular pathogenesis of “DIC” (EA-VMTD). The molecular events of “DIC”, which is endotheliopathy-associated VMTD (i.e., TTP-like syndrome) with many associated clinical organ syndromes, can be explained by expanded ULVWF path as illustrated in the following “two-activation theory of the endothelium”. For example, in sepsis complement activation occurs and attacks pathogen as a part of innate immune response. In addition to lysis of pathogen by the terminal product C5b-9, it also may induce ECs damage and endothelial dysfunction to the host if endothelial membrane is unprotected by CD59. Endotheliopathy mediates both inflammatory pathway and microthrombotic pathway. Activated inflammatory pathway promotes inflammation and activated microthrombotic pathway triggers microthrombogenesis leading to EA-VMTD if ADAMTS13 is insufficient due to unbalanced excess of ULVWF from endothelial exocytosis or due to partial ADAMTS13 deficiency from heterozygous gene mutation of ADAMTS13 gene. EA-VMTD orchestrates thrombocytopenia, MAHA, and MODS, which are the clinical features of TTP-like syndrome/“DIC”. Abbreviations: AI, adrenal insufficiency; ALF, acute liver failure; ANP, acute necrotizing pancreatitis; ARDS, acute respiratory distress syndrome; ARF, acute renal failure; “DIC”, false disseminated intravascular coagulation; DES, diffuse encephalopathic stroke; DMI, diffuse myocardial infarction; EA-VMTD, endotheliopathy-associated VMTD; DIT, disseminated intravascular microthrombosis; ECs, endothelial cells; FHF, fulminant hepatic failure; HELLPs, hemolysis - elevated liver enzymes - low platelet syndrome; MAHA, microangiopathic hemolytic anemia; SIRS, systemic inflammatory response syndrome; MODS, multiorgan dysfunction syndrome; SS, septic shock; TTP, thrombotic thrombocytopenic purpura; ULVWF, unusually large von Willebrand factor multimers

**Fig. 2:**
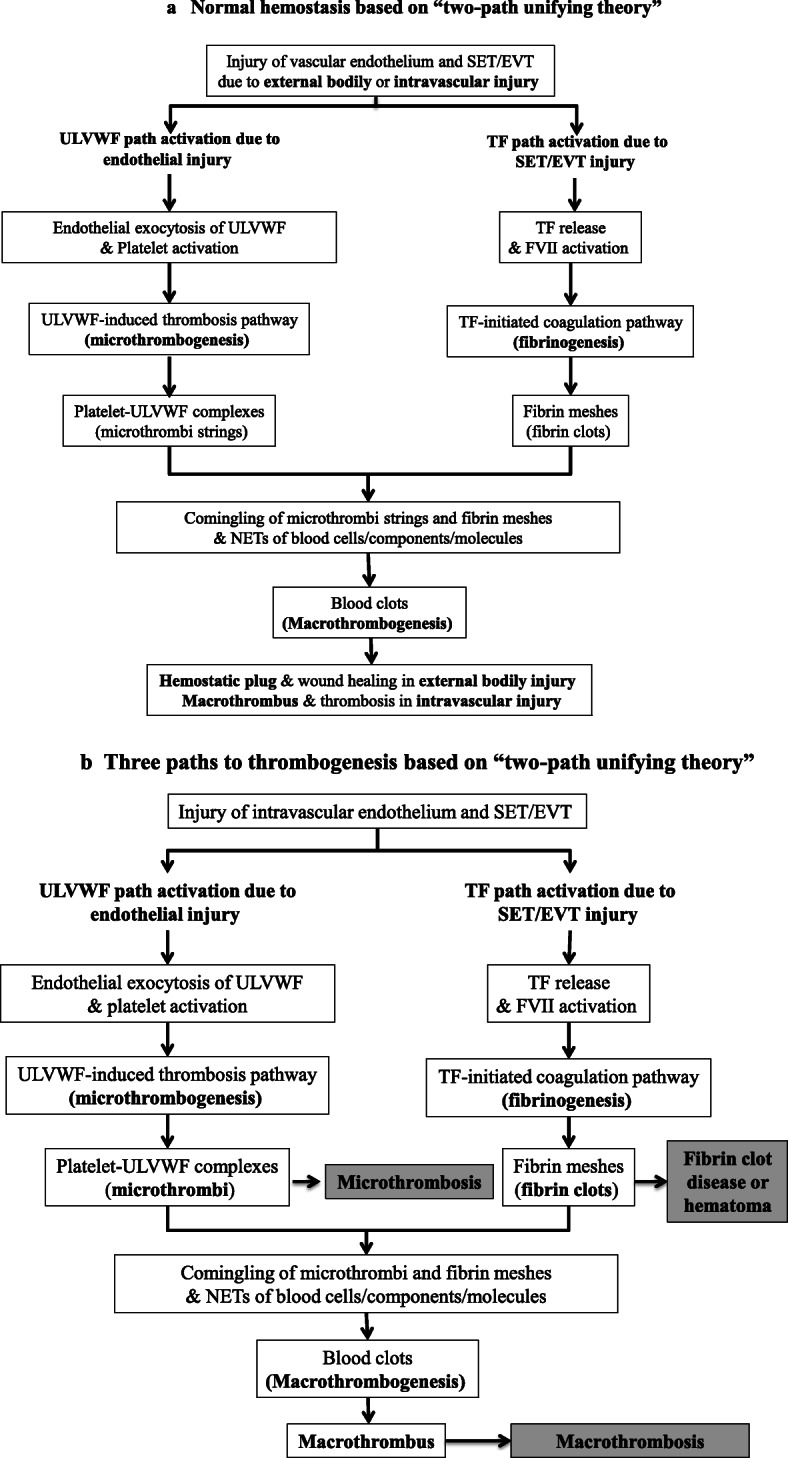
**a** Normal hemostasis based on “two-path unifying theory”. (Updated and reproduced with permission from Walters Kluwer Health, Inc., and Chang JC: Blood coagulation Fibrinolysis 2018; 29:573–584). The concept of this theory is derived from physiologic changes associated with the different levels of vascular wall damage in vascular injury as explained in three essentials in normal hemostasis (Table 3). The nature has endowed human with only “one” normal hemostatic system. Hemostasis protects lives from unnecessary bleeding in external bodily injury and aids in self-wound healing. It also warns human to avoid unnecessary self-inflicted injury and hostile environmental insult that can cause intravascular injury leading to deadly thrombotic disorders. This is true irony of nature that exactly same normal hemostasis not only provides wound healing but also can lead to life-threatening thrombosis. As explained in the text, two sub-hemostatic paths (ULVWF and TF) are initiated in vascular injury, which have to be unified to lead to normal blood clotting in external bodily injury and trigger macrothrombus in intravascular injury. In a certain disease (e.g., sepsis), only ULVWF path is activated as seen in “DIC”, and in another disease (e.g., APL), only TF path is activated as seen in true “DIC”. The former produces EA-VMTD and the latter produces fibrin clot disease. If both paths were activated simultaneously in local traumatic vascular injury, the unifying process of two hemostatic paths is called macrothrombogenesis, which produce macrothrombus. These three paths of thrombogeneses are explained in the text [3, 4]. Abbreviations: APL, acute promyelocytic leukemia; “DIC”, false disseminated intravascular coagulation; EA-VMTD, endotheliopathy-associated vascular microthrombotic disease; EVT, extravascular tissue; SET, subendothelial tissue; NETs, neutrophil extracellular traps; TF, tissue factor; ULVWF, unusually large von Willerand factor multimers. **b** Three paths to thrombogenesis based on “two-path unifying theory”. (Reproduced with permission from Walters Kluwer Health, Inc., and Chang JC: Blood coagulation Fibrinolysis 2018; 29:585–595). Figure 2b illustrates three different thrombogenetic paths in “two-path unifying theory of hemostasis” (microthrombogenesis, fibrinogenesis and macrothrombogenesis) to form respective blood clots. Each pathogenesis occurs when ULVWF path, TF path or combined path is utilized depending upon the levels of vascular wall damage following intravascular injury (ECs, SET, and EVT). The characters of the thrombi/fibrin clots from different paths are very different and produce distinctly different clinical phenotypic thrombotic disorders. In “DIC”, microthrombogenesis occurs due to lone activation of ULVWF path and leads to EA-VMTD, which hematologic phenotype is TTP-likes syndrome. Abbreviations: “DIC”, false disseminated intravascular coagulation; EA-VMTD, endotheliopathy-associated vascular microthrombotic disease; ECs, endothelial cells; EVT, extravascular tissue; SET, subendothelial tissue; NETs, neutrophil extracellular traps; TF, tissue factor; ULVWF, unusually large von Willebrand factor multimers

Recently, this author has proposed two novel theories based on vascular wall physiology and hemostasis in vascular injury; one is the molecular pathogenesis of endotheliopathy [2] illustrated in Fig. 1 to explore the mechanism of inflammation and coagulation, and the other is a in vivo hemostatic theory [3] presented in Fig. 2a, which is modeled on physiologic changes from the damage of the blood vessel wall, activating unusually large von Willebrand factor (ULVWF) path and tissue factor (TF) path in hemostasis [3, 4]. The first theory supports independent induction of inflammation and microthrombosis in generalized endotheliopathy developing in critical illnesses such as sepsis and extensive vascular trauma. The second formulates the conceptual framework of normal hemostasis and thrombogenesis in vivo*.* Together, these two theories can easily define hitherto unknown thrombogenetic mechanisms of microthrombi, fibrin clots and macrothrombus, and different clinical phenotypes of the thrombotic disorder [3, 4]. Applying these theories, the mechanism of microthrombogenesis from the role of endotheliopathy in intravascular injury is discovered and the pathogenesis of inflammation is separately identified. Further, the concepts of hemostasis and thrombogenesis are found to be congruous each other, and the genesis of different hemorrhagic diseases and phenotypes of thrombosis has been elucidated [3–6].

Following a vascular injury, macrothrombus formation occurs via normal hemostatic mechanism through two initiating paths (i.e., ULVWF path to microthromobogenesis and TF path to fibrogenesis), and subsequently through the merging path (i.e., combined path to macrothrombogenesis). The phenotypes of these three thrombo-coagulation processes may produce 1) microthrombi, 2) fibrin clots, 3) macrothrombus, or 4) hematoma [3, 4]. The analytical study of thrombogenetic mechanism on the current concept of hemostasis can prove that DIC observed in sepsis, which has been ascribed to TF-initiated fibrinogenesis, is incorrect. Instead, DIC occurs as a result of microthrombogenesis due to activation of lone ULVWF path of hemostasis. DIC is characterized by “microthrombi” occurring in endotheliopathy-associated vascular microthrombotic disease (EA-VMTD) [3–8].

In this review, this author will briefly look back the history of DIC controversy, and present additional supporting evidence for the new identity of “DIC” since a previous publication of contentious debate on DIC [5]. Since the comprehension of true in vivo hemostasis is the only way to resolve current controversy through explanation of the pathophysiological mechanism of DIC, the discussion of this article will mainly be devoted to reviewing the physiological mechanism of normal and pathologic hemostasis from the physical changes associated with the extent of vascular wall damage and explaining two theories of in vivo hemostasis and molecular endothelial pathogenesis. The quotation mark on “DIC” has been placed to indicate current term DIC is incorrect and its concept is falsely founded. In the rest of this article, both the term “DIC” and false DIC will be used interchangeably.

## A historical reflection

### Thrombotic thrombocytopenic purpura and “disseminated intravascular coagulation”

In 1924, Moschcowitz [9] first recognized a thrombotic blood disorder characterized by disseminated hyaline microthrombi in terminal arterioles and capillaries of many organs of a young woman who died of multiorgan failure. Later, Singer et al. [10] named this disorder as thrombotic thrombocytopenic purpura (TTP) and attributed it to generalized platelet thrombosis. Later, TTP was found to be caused by microthrombosis. TTP is characterized by thrombocytopenia, microangiopathic hemolytic anemia (MAHA) and often with dysfunction of the brain and kidneys. A quarter century later in 1950, McKay [1, 11] separately coined the term DIC for another blood disorder observed in a woman who died of disseminated microvascular thrombosis associated with hemorrhage progressing to multiorgan dysfunction syndrome (MODS). Because of thrombosis (coagulation) and hemorrhagic nature, the condition was termed “disseminated intravascular coagulation”. In retrospect, “DIC” ascribed by McKay and his late followers was very similar to TTP described by Moschcowitz in its pathologic feature of disseminated intravascular microthrombosis (DIT) and hematologic features of thrombocytopenia and MAHA as well as organ dysfunction syndrome.

“DIC” typically has occurred in association with critical illnesses such as sepsis and polytrauma, and often accompanied by hemorrhagic syndrome. The hemorrhagic syndrome has been inferred to be due to “consumption coagulopathy” resulting from disseminated fibrin clots formation and consumption of coagulation factors. Since acute promyelocytic leukemia (APL) is also characterized by consumption coagulopathy, this condition has been included in the column of “DIC” although hematologic features are very different from “DIC”. Several decades ago, clinical pathologists [12, 13] have found the pathology of “DIC” was consistent with “microthrombi” very similar to those of TTP, which are pathologically different from “fibrin clots” seen in DIC of APL. However, many coagulation specialists have used the terms “microthrombi” and “fibrin clots” interchangeably even though the true character of DIC of APL is made of fibrin clots and that of “DIC” observed in sepsis is made of microthrombi composed of platelet-ULVWF complexes, which character is the same to microthrombi occurring in TTP [14]. This interchangeable interpretation simply has reflected the ironclad concept that all “blood clots” (i.e., thrombi and fibrin clots) must be the same because the process of hemostasis and its end product of coagulation must be the same.

Until this date, in clinical settings, “DIC” and TTP are considered to be two distinctly different microthrombotic disorders, somehow each arising from utilization of yet undetermined but the same pathogenetic mechanism because “DIC” is clearly a hemostatic disorder causing hemorrhagic syndrome associated with conditions such as sepsis, and TTP is an enzymatic disorder due to ADAMTS13 deficiency. In addition, TTP-like syndrome has oftentimes been included in the column of TTP, and was called “thrombotic microangiopathy”, which term does not represent a disease [6]. Currently, the “thrombotic microangiopathy” has been designated when thrombocytopenia and MAHA are present without severe ADAMTS13 deficiency in an acquired medical condition. Nonetheless, pathologically all of them are characterized by microthrombosis in arterioles and capillaries. When sepsis presents with clinical features of thrombocytopenia, MAHA, MODS and abnormal coagulation profile, most of clinicians have called it “DIC”, but others classified it as TTP-like syndrome when coagulation profile was normal [6, 15]. Some theorists have insisted that “DIC” and “TTP-like syndrome only share the same pathophysiological mechanism because of different underlying diseases [16]. However, this author has affirmed “DIC” and TTP-like syndrome are the same disease after clinical, pathological, hematological and coagulation evaluation as well as atypical behavior of “DIC” to numerous anticoagulation therapies [5, 6, 17]. Previously, the conceptual difference between TTP and “DIC” was supported by the claim that TTP was due to excess of ULVWF as the result of protease ADAMTS13 deficiency, but “DIC” was due to a hemostatic disease associated with endothelial dysfunction as seen in sepsis. TTP is defined as a microthrombotic disease associated with ADAMTS13 deficiency resulting from either hereditary TTP (gene mutation-associated vascular microthrombotic disease [GA-VMTD]) or due to acquired autoimmune TTP (antibody-associated VMTD [AA-VMTD]). But conceptually “DIC” has been attributed to uncontrolled activation of disseminated intravascular hemostasis, leading to fibrin clot disease via activated TF path, which is only known thrombotic (coagulation) path in hemostasis at this time. Therefore, abnormal coagulation profile in “DIC” is thought to be the result of consumption and depletion of clotting factors during disseminated intravascular coagulation and secondary fibrinolysis [16, 18, 19].

Understandably, contemporary dogmatic hemostatic theory based on TF path, which is only one pathway known in hemostasis in vivo, has been applied to the interpretation of “DIC”. It is defined to be coagulopathy caused by endotheliopathy in sepsis and other critical illnesses, triggering TF/FVIIa-initiated coagulation cascade that mediates systemic inflammation and coagulation/thrombosis, leading to blood clots containing fibrins. Because of coexisting inflammation and coagulopathy in endotheliopathy, the popular theory of “crosstalk” between inflammation and coagulation has been introduced and readily accepted. Also, the use of different descriptive terms of “thrombosis” on TTP/TTP-like syndrome and “coagulation” on “DIC” in the medical literature has kept the distance between two diseases even though their underlying pathology is the same “microthrombosis”. For TTP/TTP-like syndrome and “DIC”, more generalized character term “microvascular thrombosis” has been designated and accepted to be due to ADAMTS13 deficiency for the former (TTP) and endotheliopathy for the latter (“DIC”). But for “DIC”, the term “coagulation” is used and has implied that “DIC” is “fibrin clot disease” occurring as a result of uncontrolled hemostasis. These designations have strengthened the notion that TTP, TTP-like syndrome and “DIC” are three different diseases. TTP is an enzymatic disease, TTP-like syndrome is a condition associated with “thrombotic microangiopathy”, and “DIC” is a hemostatic disease. This misconception that fibrin clots are responsible for “DIC” has contributed to the delay for the medical community to recognize the genuine character of “DIC” and discovering the true mechanism of in vivo hemostasis.

Sometimes clinicians have been puzzled and fascinated by the “shared” pathogenetic mechanism not only among TTP, TTP-like syndrome and “DIC”, but also other hematologic disorders producing thrombocytopenia and MAHA, MODS such as poorly defined thrombotic microangiopathy, and hemolytic-uremic syndrome (HUS) [16, 18–21]. However, these ambiguous concepts between “microvascular thrombosis” and “coagulation” have never been seriously questioned and explored, primarily due to unmistakably characteristic feature of hemorrhagic syndrome observed in acute “DIC”.

### Chronic “disseminated intravascular coagulation” without coagulopathy

Not surprisingly, another conflicting concept about “DIC” has begun to encroach into the critical care medicine where the use of the term “DIC” in clinical diagnosis has been liberalized and expanded to include the cases of poorly defined “thrombotic microangiopathy” even with normal coagulation profile and absence of hemorrhagic syndrome [22, 23]. Many critical care clinicians have become comfortable in taking this proposition as chronic “DIC” whenever thrombocytopenia, MAHA and MODS in particular occur in critically ill patients even though no hemorrhagic syndrome is presented. Some coagulation specialists have been reluctant to use this identity as “chronic DIC”. In Japan, this additional term has been very enthusiastically accepted even with governmental funding and supports and is now designated as “chronic DIC”. European hemostasis/thrombosis community also has used these terms extensively, including “compensated” or “covert” “DIC” in contrast to “acute”, “uncompensated”, or “overt” “DIC” when significant hemorrhagic phenotype occurs.

Yet, some hematologists have stubbornly insisted the term TTP or TTP-like syndrome for coagulopathy-negative “DIC”. Furthermore, additional notable encounter was the perplexity of uncommon organ phenotypes in MODS such as acute renal failure, acute necrotizing pancreatitis, acute respiratory distress syndrome, fulminant hepatic failure, hepatic encephalopathy, cardio-pulmonary syndrome, hepato-renal syndrome, rhabdomyolysis, adrenal insufficiency, microvascular myocardial infarction and others [6, 24], which often have occurred in association with thrombocytopenia and MAHA without gene mutation of hereditary TTP or antibody production of acquired TTP. Some have called it TTP-like syndrome [6] and others used the term atypical acquired TTP, hemolytic-uremic syndrome, or thrombotic microangiopathy. Indeed, in clinical practice, EA-VMTD (i.e., TTP-like syndrome) is much more common disorder than GA-VMTD and AA-VMTD. Now, every TTP-like syndrome can be classified as EA-VMTD based on entirely different endothelial molecular pathogenesis from classical TTP that is caused by severe ADAMTS13 deficiency [6].

Over the years, the enigmatic clinical features expressed by microthrombosis have aroused a concern in the mind of some clinicians, not only about the underlying disease, pathogenesis, diagnostic tests and treatment approach, but also about different clinical identity and phenotype amongst acute “DIC”/chronic “DIC”, TTP/TTP-like syndrome, HUS/acute renal failure, thrombotic microangiopathy/microvascular thrombosis, and MODS/biorgan syndrome [6, 16–24]. Although TTP/TTP-like syndrome and “DIC”/DIT appear to be different in clinical phenotypes, the character of microthrombi is pathologically the same and microthrmbi are composed of platelet-ULVWF complexes [5, 14]. Thus, the term VMTD has been introduced to encompass the entire spectrum of “microvascular thrombosis” and “vascular microthrombosis” to include focal, multifocal, local, regional and disseminated VMTD [2–6]. How ironical it is to note that the etiology, pathogenesis, diagnostic test and treatment are well established in TTP, which underlying pathology is VMTD, but we know nothing about the above clinical parameters in “DIC”, which underling pathology is also VMTD. The true mechanism of microthrombogenesis [2] causing “DIC” has eluded the scientists’ intellect in medical science and the clinician’s acumen of practice in medicine until this date. No doubt, it should be blamed to the fact that our understanding of in vivo hemostasis and thrombogenesis is not completely identified yet [3, 4].

## Lessons learned from “disseminated intravascular coagulation” controversy

### Unsettled theories for the pathogenesis of “disseminated intravascular coagulation”

When we look back the medical history since its initial description of “DIC” based on ill-founded concept, the medical literature is replete with published articles on the enigmatic nature of the pathophysiological mechanism of “DIC” from many bright minds of coagulation science and clinical medicine [25–39]. Numerous theories had and has been proposed to explain the pathogenesis of “DIC” based on the mechanism of coagulation, which includes nitrogen bubble induction [26], cerebral ischemia [29], TF/FVIIa-initiated coagulation cascade [25, 30], cross-talk between inflammation and coagulation [30–36], glycocalyx degradation [33], FXII-initiated consumption coagulopathy [34], neutrophil extracellular traps (NET)-induced immunothrombosis [35–37], shock-induced endotheliopathy [38], brain regulation [39] and many others, but none of them has convincingly explained the unique clinical, laboratory and pathological features of “DIC”.

In retrospect, it is obvious that the incomprehensible nature of microthrombosis/fibrin clot disease has been due to yet incompletely identified in vivo hemostatic mechanism. Because of this limitation, clinicians and coagulation scientists have diverted their research efforts to look for the pathogenetic mechanism linking between “DIC” and microthrombosis, and have begun to apply new approaches. Science have moved away from the hemostatic model of the physiology of vascular system to molecular biological mechanisms of cellular/organ system dysfunction within immune system [35–37], endocrine system [38], central nervous system [39], and even neuronal cells [29] to look for yet unknown complex molecular signal mechanisms. But, identifying the molecular pathophysiological mechanism of microthrombogenesis has been unsuccessful.

Recently, the research efforts have intensively focused on the role of neutrophil extracellular traps in hemostasis, identifying intravascular traps of circulating apoptotic blood cells and molecules within the vascular system, with enthusiasm and hope. The theory of immunothrombosis supported by NETosis and molecules from blood cell components has attracted many molecular scientists in search for the pathogenesis of thrombosis and microthrombosis. Hemostasis has been considered to be natural defensive mechanism against pathogen via its critical role utilizing cellular and molecular traps derived from immune cells as well as blood cells. However, the first essential principle that hemostasis and thrombosis must be activated only in vascular injury cannot be juxtaposed with immunothrombosis in both physiologic and pathologic hemostasis [3, 17]. The theory of NETosis is not consistent with this hemostatic fundamental because, without intravascular damage, tissue factor (TF) as well as ULVWF cannot be released to activate blood coagulation, and thrombosis could not be formed. No doubt, the key mechanism of in vivo hemostasis is determined by the response of vascular wall physiology following intravascular injury [3, 4, 17]. NETs cannot be the initiator of hemostasis, but happens to be the innocent bystander being trapped passively within the clots or perhaps secondary participant actively involved in trapping themselves within thrombogenetic scaffolds with a purpose [17]. NETosis is likely an auxiliary process only after initiation of hemostasis following intravascular injury. There are voices among molecular scientists cautioning the primary role of immunothrombosis in thrombogenesis [36, 37, 40, 41].

### Coagulation and thrombogenesis (fibrin clots vs. microthrombi)

To date, the term “coagulation” is used to denote a “fibrin clots” forming process either in vitro and in vivo, which can be more precisely termed as “fibrinogenesis”. Fibrinogenesis can be defined as in vitro fibrin forming process via extrinsic cascade in the test tube and also a part of in vivo blood clotting process of hemostasis in vascular injury. Coagulation is conceptually interpreted as a process generating blood clots (i.e., primarily made of fibrin clots and blood cells) seen such conditions as deep vein thrombosis (DVT), pulmonary embolism and acute ischemic stroke. Coagulation community has been in general agreement with the conception that hemostasis is the same blood clotting process in both bleeding control of external bodily injury, leading to coagulation, and blood clot forming process (i.e., thrombogenesis) in intravascular injury, leading to thrombosis. However, a major dilemma is we cannot reconcile the different role of various coagulation participants in coagulation and thrombogenesis. This has been particularly true for ULVWF, which we know, is the main component interacting with the platelet and initiating in vivo coagulation via platelet plug formation in external bodily injury. The precise role of ULVWF, however, has not been identified in thrombogenesis in intravascular injury although it is suspected to play a significant role [42, 43]. To make the matters even more difficult, we have no clear understanding of the difference in the concept between coagulation and thrombogenesis in vivo for the role of interaction between ULVWF and fibrins.

One question this author likes to ask is why McKay coined the term “DIC” instead of “disseminated intravascular thrombosis (DIT)”. In the lexicon of hemostasis, we call every intravascular blood clotting disorder as “thrombosis”, including DVT, TTP/TTP-like syndrome and heparin-induced thrombocytopenia with thrombosis, but the term “coagulation” is applied only in “DIC” for a disease designation. Traditionally the term “coagulation” has not been used in the describing a disease in each and every thrombotic disorder. From the start, this author believes the term “DIC” was incorrectly designated and has contributed false conceptualization of its pathogenesis.

Another concern is the character description of “blood clots” is imprecise although it carries the inclusive and perceptive meanings of “thrombus”, “microthrombi”, “fibrin clots”, and “hematoma” and supposedly represents more or less similar composites within their clots because, in current theory, all blood coagulation begins with activation of TF/FVIIa path and ends with “blood clots” composed of fibrin meshes, platelets, cellular traps and molecules, and other blood cells. From this proposition, the therapeutic approach has exploited the belief that the same hemostatic mechanism in the management of various thrombotic/coagulation disorders requires the same anticoagulation in every thrombosis and clot disease, and every clinical trial, but this therapeutic effort did not work well in “DIC” and hemorrhagic disease of APL as well as TTP-like syndrome with coagulopathy. Another approach was the counteracting therapy against expressed TF path biomarkers such as antithrombin, activated protein C, tissue factor pathway inhibitor and thrombomodulin. So far, every venture has shown no clear benefit in therapeutic trials.

When we look back these failures, it is no wonder why these approaches were not successful in sepsis-associated “DIC” and other microthrombotic disorders. It is because the characters of “microthrombi” composed of platelet-ULVWF complexes are very different from “macrothrombus” made of platelet and fibrin clots as well as blood cells and their components. On the one hand, microthrombi in sepsis are produced via activation of lone ULVWF path promoted by generalized exocytosis of ULVWF from damaged endothelial cells (ECs) in endotheliopathy (Figs. 1 and 2a) [5, 6]. On the other hand, macrothrombus in intravascular trauma (e.g., vascular procedures) is produced via combined activation of ULVWF path and TF path triggered by local release of ULVWF and TF from damaged ECs and subendothelial tissue (SET)/extravascular tissue (EVT) in intravascular injury [4, 17]. Here, vascular wall physiology plays a critical role in thrombogenesis in intravascular injury as well as hemostasis in external bodily injury.

The existence of these two different characters of “microthrombi” and “macrothrombus” had alerted this author that two different hemostatic paths must be present in vivo; one must be the result of activated ULVWF path and the other is that of activated TF path [3, 4]. Microthrombi must be promoted by microthrombogenesis, which is ULVWF-initiated thrombogenesis with recruitment of platelets. Additionally, fibrin clots are produced by fibrinogenesis, which represents TF/FVIIa- initiated coagulation cascade. Further, to make a complete form of macrothrombus, these two incomplete products, microthrombi made of platelet-ULVWF complexes and fibrin clots made of fibrin meshes, must be unified via macrothrombogenesis to produce macrothrombus [3, 4]. Based on this analysis and logic, this author has envisioned the framework of in vivo hemostasis and proposed that, in addition to currently known coagulation (TF) path, another path must exist, which can be called microthrombotic (ULVWF) path. These insights through the reinterpretation of false DIC have led for this author to identify novel “two-path unifying theory” of hemostasis and this proposal has given us a birth of complete picture of in vivo hemostasis in the context of vascular wall physiology, which can explicate every hemorrhagic disease and every thrombotic disorder.

### Hemorrhagic disease of acute “DIC” and importance of FVIII/VWF

Whenever abnormal coagulation profile is encountered in a critically ill patient, the differential diagnosis should include 1) acute “DIC”, 2) EA-VMTD with hepatic coagulopathy, and 3) primary fibrinolysis because they often present with thrombocytopenia, hyperfibrinogenemia or hypofibrinogenemia, prolonged prothrombin time and activated thromboplastin time, and elevated D-dimer. The critically ill patients with microthrombosis (i.e., EA-VMTD) are always characterized by markedly increased activity of FVIII and increased expression of VWF/ULVWF/VWF antigen following endothelial exocytosis of ULVWF/FVIII [44–47]. Hyperfibrinogenemia in early phase of EA-VMTD-associated hepatic coagulopathy is due to release of fibrinogen from damaged hepatocytes. These findings rule out acute “DIC” as a correct diagnosis because it, according the concept of consumption coagulopathy, must be characterized by markedly decreased FVIII, decreased VWF and hypofibrinogenemia (Tables 1 and 2). Also, primary fibrinolysis can be ruled out due to the laboratory findings of hyperfibinogenemia and increased activity of FVIII, which should be decreased in fibrinolysis. The coagulation parameters of markedly increased activity of FVIII and increased expression of VWF/ULVWF/VWF antigen can be easily and perfectly reconciled with endotheliopathy-associated microthrombosis (i.e., EA-VMTD). Mild to moderately elevated D-dimer supports microthrombosis-induced liver cell damage (e.g., hepatic fibrinogenolysis) following activated ULVWF path rather than primary role of activated TF path. This differential diagnosis will be further discussed later in this article.

**Table 1:** Hematologic differential diagnosis amongst VMTD and similar coagulopathies

	GA-VMTD (hereditary TTP) AA-VMTD (autoimmune TTP)	EA-VMTD without HC (chronic “DIC”)	EA-VMTD with HC due to acute liver necrosis (acute “DIC”)	Fibrin clot disease (True DIC) (e.g., APL)	Primary fibrinolysis (e.g., amyloidosis)
**Hematologic features**					
Thrombosis form	Microthrombi	Microthrombi	Microthrombi	Fibrin clots	NA
Hemostatic path	Aberrant ULVWF path	ULVWF path	ULVWF path	Aberrant TF path	NA
Thrombocytopenia	Always present	Variably present	Commonly present	Present due to APL	Not present
MAHA	Always present	Commonly present	Commonly present	Not present	Not present
MODS	Often present (brain and kidneys)	Often present	Often present	Not present	Not present
Acute liver failure	Uncommon (?)	Not present	Always present	Not present	?
Hemorrhage	Typically do not occur	Do not occur	Typically occur	Commonly occur	Persistent bleeding
**Coagulation factors**					
Fibrinogen	Normal	Normal	Increased (early phase) decreased (late phase)	Decreased	Markedly decreased
FVIII	Normal	Markedly increased	Markedly increased	Markedly decreased	Decreased
FV	Normal	Normal	Decreased	Decreased	Normal to decreased
FVII	Normal	Normal	Markedly decreased	Normal	Normal
D-dimer/FDP	Normal	Normal	Increased	Increased	Increased
VWF/ULVWF	Normal	Markedly increased	Markedly increased	?	?
**Coagulation tests**					
aPTT	Normal	Normal	Markedly prolonged	Markedly prolonged	Prolonged
PT	Normal	Normal	Markedly prolonged	Prolonged	Prolonged

Abbreviations: *APL* Acute promyelocytic leukemia, *DIC* Disseminated intravascular coagulation, *DIT* Disseminated intravascular microthrombosis, *FDP* Fibrin degradation products, *MAHA* Microangiopathic hemolytic anemia, *aMAHA* Atypical MAHA, *MODS* Multiorgan dysfunction syndrome, *aPTT* Activated partial thromboplastin time, *PT* Prothrombin time, *TF* Tissue factor, *TT* Thrombin time, *TTP* Thrombotic thrombocytopenic purpura, *VWF* Von Willebrand factor multimers, *ULVWF* Unusually large von Willebrand factor multimers, *VMTD* Vascular microthrombotic disease, *AA-VMTD* Antibody-associated VMTD, *EA-VMTD* Endotheliopathy-associated VMTD, *GA-VMTD* Gene mutation-associated VMTD

**Table 2:** Hemostatic characteristics and mechanisms between “DIC”/DIC and EA-VMTD-associated hepatic coagulopathy

	Acute “DIC” (per current concept*)	EA-VMTD-associated hepatic coagulopathy	DIC of APL
**Clinical settings**	Critical illnesses (e.g., sepsis; trauma)	Critical illnesses (e.g., sepsis; trauma)	APL
**Thrombosis form**	Claim to be fibrin clots* by some, but are microthrombi by pathology	Microthrombi	Fibrin clots
**Pathogenesis**	Fibrinogenesis*	Microthrombogenesis	Fibrinogenesis
**Hemostatic path**	TF path activation*	Lone activation of ULVWF path	Aberrant TF path activation
**Coagulation study**			
Platelet count	Low	Often low	Low (due to leukemia)
PT	Prolonged	Prolonged	Prolonged
aPTT	Prolonged	Prolonged	Prolonged
FVIII	Supposedly markedly ↓ (due to consumption)	Markedly ↑ (due to endothelial release)	Markedly ↓ (due to consumption)
FV	Supposedly ↓ (due to consumption)	Moderately ↓ (due to live necrosis)	Decreased (due to consumption)
FVII	Supposedly normal (because not consumed)	Markedly ↓ (due to liver disease)	Normal
Fibrinogen	Supposedly ↓ (due to consumption)	Markedly ↑ (due to release in early liver damage) Markedly ↓ (due to liver failure in late stage)	Decreased (due to consumption)
ULVWF/VWF	Supposedly normal (due to no participation)	Markedly ↑ (due to endothelial exocytosis)	Negative (due to no endotheliopathy)
D-dimer/FDP	Positive (due to “fibrinolysis*”)	Positive (due to fibrinogenolysis in liver damage)	Positive (due to fibrinolysis)
**Clinical syndrome**	Supposedly consumption coagulopathy*, but this disease with the above Lab findings does not exist in real patient. (Please see text for COVID-19 interpretation)	EA-VMTD (i.e., DIT) with hepatic coagulopathy	Consumption coagulopathy of APL
**Correct diagnosis**	Does not exist, but it should be → → →→	EA-VMTD with HC	True DIC (fibrin clot disease)

*Abbreviations*: *APL* Acute promyelocytic leukemia, *DIC* Disseminated intravascular coagulation, *“DIC”* False DIC, *DIT* Disseminated intravascular microthrombosis, *EA-VMTD* Endotheliopathy-associated vascular microthrombotic disease, *FDP* Fibrin degradation products, *HC* Hepatic coagulopathy, *TF* Tissue factor, *TTP* Thrombotic thrombocytopenic purpura

*Wrongfully designated pathogenesis and concept

Additionally, unlike false DIC, “true DIC” (e.g., APL) shows the feature of consumption coagulopathy, which includes decreased fibrinogen level, and decreased FVIII and FV activity because fibrinogen is consumed, and FVIIIa and FVa become inactivated after modulation via activated TF-induced fibrinogenetic path. Strangely, in coagulation study of acute “DIC” in “real” patients, the activity of FVIII was markedly increased and VWF/ULVWF overexpressed [5, 44–47]. These findings alerted this author to question the correctness of the concept of “DIC”. Certainly, the diagnosis of the coagulopathy based on changes in coagulation factors in acute “DIC” patients was more consistent with EA-VMTD with hepatic coagulopathy due to microthrombosis-induced liver necrosis. These laboratory findings summarized in Tables 1 and 2 are congruous with the concept that “DIC” and EA-VMTD are the same disease and support the underlying pathology is microthrombosis. This clarification on the role FVIII and ULVWF/VWF in microvascular thrombosis has corrected the major misinterpretation between the supposedly hemorrhagic disease of acute DIC and hepatic coagulopathy in EA-VMTD-induced liver necrosis [44–50]. This error seems to be a trivial one, but this minor misrepresentation in medical community has hugely changed the history of medicine and contributed to the delay in identifying the mechanism of in vivo hemostasis.

The mechanism of alteration of coagulation factors between “true” DIC (i.e., APL) and false acute “DIC” are separately summarized with EA-VMTD-associated hepatic coagulopathy in Table 2 with explanation. Unlike EA-VMTD, consumption coagulopathy of APL with decreased activity of fibrinogen, FVIII and FV can be affirmed to be true DIC (fibrin clot disease) characterized by disseminated fibrin clots. The decreased coagulation factors in APL are the result of consumption of fibrinogen, and consumption and inactivation of FVIIIa and FVa. Increased D-dimer/FDP is the result of secondary fibrinolysis.

Markedly increased activity of FVIII and overexpressed VWF/ULVWF in patients presenting with EA-VMTD (e.g., TTP-like syndrome) have been mistakenly interpreted to be “hypercoagulable state” or “thrombophilic state” that causes thrombosis or coagulopathy. Instead, the correct interpretation is those changes are the result of endothelial exocytosis in EA-VMTD triggered by endotheliopathy. Better understanding for the role of ULVWF path and TF path in hemostasis in vivo should clarify many unaccountable “poorly defined coagulopathy”, “DIC-like syndrome” and “dysregulated coagulation system” in patients with thrombotic disorder and hemorrhagic disease. If a critically ill patient shows markedly increased FVIII activity and increased expression of VWF/ULVWF, please look for “masked” EA-VMTD and potential diagnosis of TTP-like syndrome.

### Crosstalk between inflammation and coagulation

Frequent coexistence of inflammation and coagulation disorder in sepsis and critical illnesses put forward the crosstalk theory to tie inflammation to microthrombosis [51–53], but this interaction theory cannot explain the difference of molecular mechanism producing microthrombi and macrothrombi, and is unable to support the concept of in vivo hemostasis via cellular and vascular physiology occurring in intravascular injury. When sepsis progresses to endotheliopathy, both inflammatory pathway and microthrombotic pathway are activated simultaneously but independently as presented in “two-activation theory of the endothelium” (Fig. 1) [2]. Endothelial dysfunction promotes inflammation mediated by cytokines released from ECs and microthrombogenesis initiated by exocytosis of ULVWF from Weibel-Palade bodies located within ECs. However, no research has shown how cytokines alter hemostatic components ULVWF and TF that triggers to thrombogenesis. In sepsis and critical illnesses, clinical features of inflammation such as fever, malaise, myalgia and arthralgia, and those of coagulopathy such as microthrombosis and hemorrhagic syndrome can be better explained by each independent pathogenesis [2, 7]. Inflammation is not the cause of endotheliopathy, but is the result of endothelial dysfunction, which led to the release of various cytokines [17]. Even though inflammation and vascular microthrombosis often coexist together in endotheliopathy, inflammation has not shown to promote the activation of hemostasis components ULVWF or TF, nor acute and chronic inflammatory conditions are known to enhance hemostasis without endotheliopathy.

Additionally, it should be noted that inflammation does not occur in AA-VMTD (i.e., TTP) even though platelet-ULVWF complexes are the same microthrombi produced in EA-VMTD [17]. This observation also supports inflammation is primarily the result of endotheliopathy, and is not the essential component leading to coagulation or microthrombogenesis. Further, antiinflammatory agents targeting the inflammatory cascade have failed in clinical trials for sepsis-associated coagulopathy.

### Altered expression of tissue factor path markers

Until now, the pathogenesis “DIC” has been based on the ironclad concept of activated TF path forming “fibrin clots”. This notion has been further solidified in sepsis by altered expression of TF path markers as well as antithrombotic and prothrombotic endothelial markers. These include thrombin, antithrombin, thrombin-antithrombin complex, activated protein C (APC), tissue factor pathway inhibitor, thrombomodulin, and D-dimer [54, 55]. Even though altered TF path markers indirectly seem to support the role of activated TF path in “DIC”, these findings can be better explained by alternative mechanism of expression from the subendothelial tissue (SET) damage of vascular walls and extravascular tissue (EVT) damage associated with organ dysfunction. This tissue damage occurs due to microthrombi-induced hepatic necrosis in the liver and other organ damage, and also due to vascular injury resulting from surgery and vascular access procedures in the hospitalized patient. The SET/EVT damage would release TF and alter the expression of TF path related markers. Thus, altered TF path markers in “DIC” are suspected to be the result of secondary event that are unrelated to primary pathogenesis of activated ULVWF path. This complexity of various hemostatic path markers also has contributed to the misinterpretation of sepsis-associated coagulopathy.

The prevention of “DIC” in septic patients should be a key therapeutic target in reducing death from multisystem organ failure [56]. To date, no effective therapy for sepsis and septic shock has been found to reverse advanced MODS after many decades of labor intensive and highly expensive clinical trials based on contemporary theory of TF initiated hemostasis [57–59]. Should we blame the failure of clinical trials to the intricacy of sepsis or lack of therapeutic advancement? This author can ascertain that our incomplete understanding of in vivo hemostasis was the main culprit of therapeutic failures [17]. To consider the above dilemma in perspective, in vivo hemostasis based on “two-path unifying theory” that is centered on vascular wall physiology will be briefly summarized as follows.

## Basics of hemostasis

### Vascular wall physiology and hemostatic logic in vascular injury

Nature has endowed human with only one normal hemostatic mechanism to be available for bleeding control in both external bodily injury and intravascular injury. Although hemostasis in external bodily injury protects human lives from life-threatening blood loss, it also creates thrombosis in intravascular injury harmful to human as shown in Fig. 2a and b [3].

The most important task of hemostasis is the nature’s law to sustain life through its activation in external bodily injury. However, an event of intravascular injury may damage to ECs which initiates exocytosis of ULVWF and recruit platelets, and activates ULVWF path of hemostasis. It may cause additional SET/EVT damage that releases TF into circulation and induces activation of FVII, and activates TF path of hemostasis. For example, sepsis typically causes limited but generalized damage to ECs via complement activation and pathogen-originated ligand adhesion to an endothelial cell receptor. It provokes disseminated endotheliopathy, which leads to lone activation of ULVWF and triggers microthrombogenesis without activation of TF path. However, severe local injury, vascular disease or surgery may cause not only ECs damage, but also SET/EVT damage that releases TF. It provokes activation of both ULVWF and TF paths, which together leads to macrothrombogenesis (Fig. 2b). Therefore, sepsis mediates disseminated microthrombosis (e.g., DIT; “DIC”), but extensive vascular wall injury produces local macrothrombosis (e.g., DVT, acute ischemic stroke) [60]. This simple physiologic concept of the vascular wall at different levels of damage succinctly defines the difference of the character between “DIC” and DVT. The only question to be identified is how microthrombosis and fibrin clots come together to form macrothrombus.

### Hemostatic fundamentals

#### Three essentials in normal hemostasis

The fundamental aspect of normal hemostasis and thrombogenesis is summarized in Table 3 as follows:

- ***Hemostatic principles***

**Table 3:** Three essentials in normal hemostasis

**(1) Hemostatic principles**		
(1) Hemostasis can be activated only by vascular injury.		
(2) Hemostasis must be activated through ULVWF path and/or TF path.		
(3) Hemostasis is the same process in both hemorrhage and thrombosis.		
(4) Hemostasis is the same process in both arterial thrombosis and venous thrombosis.		
(5) Level of vascular damage (ECs/SET/EVT) determines different clinical phenotypes of hemorrhagic disease and thrombotic disorder.		
**(2) Major participating components**		
**Components**	**Origin**	**Mechanism involved**
(1) ECs/SET/EVT	Blood vessel wall/EVT	Protective barrier in hemostasis
(2) ULVWF	ECs	Endothelial exocytosis; microthrombogenesis
(3) Platelets	Circulation	Adhesion to ULVWF strings; microthrombogenesis
(4) TF	SET and EVT	Release from tissue due to vascular injury; fibrinogenesis
(5) Coagulation factors	Circulation	Activation of coagulation factors; fibrinogenesis
**(3) Vascular injury and hemostatic phenotypes**		
**Injury-induced damage**	**Involved hemostatic path**	**Level of intravascular injury and thrombosis phenotypes**
(1) ECs	ULVWF	Level 1 damage – microthrombosis (e.g., TIA [focal]; Heyde syndrome [local]; EA-VMTD/“DIC” [disseminated])
(2) ECs/SET	ULVWF + sTF	Level 2 damage – macrothrombosis (e.g., AIS; DVT; PE; AA)
(3) ECs/SET/EVT	ULVWF + eTF	Level 3 damage – macrothrombosis with hemorrhage (e.g., THS; HMI)
(4) EVT alone	eTF	Level E damage – fibrin clot disease (e.g., AHS [e.g., SDH; EDH]; ICH; organ/tissue hematoma)
**Hemostatic phenotypes**	**Causes**	**Genesis of phenotypes**
(1) Hemorrhage	External bodily injury	Trauma-induced external bleeding (e.g., accident; assault; self-inflicted injury)
(2) Hematoma	Internal EVT injury	Obtuse trauma-induced bleeding (e.g., tissue and cavitary hematoma; hemarthrosis)
(3) Thrombosis	Intravascular injury	Intravascular injury (e.g., atherosclerosis; indwelling vascular device; surgery; procedure)

*Abbreviations: AA* Aortic aneurysm, *AIS* Acute ischemic stroke, *AHS* Acute hemorrhagic syndrome, ““*DIC*” False disseminated intravascular coagulation, *DVT* Deep vein thrombosis, *ECs* Endothelial cells, *EDH* Epidural hematoma, *EVT* Extravascular tissue, *HMI* Hemorrhagic myocardial infarction, *ICH* Intracerebral hemorrhage, *PE* Pulmonary embolism, *SDH* Subdural hematoma, *SET* Subendothelial tissue, *TF* Tissue factor, *eTF* Extravascular TF, *sTF* Subendothelial TF, *THS* Thrombo-hemorrhagic stroke, *TIA* Transient ischemic attack, *ULVWF* Unusually von Willebrand factor multimers, *VMTD* Vascular microthrombotic disease, *EA-VMTD/DIT* Endotheliopathy-associated vascular microthrombotic disease;

Five unwavering hemostatic principles are shown in Table 3-(1) [3, 4]. It is derived from common logics of the mechanism of hemostasis. Hemostasis in both bleeding in external bodily injury and every thrombosis in intravascular injury should confirm these five principles without consideration of the involved vessel size and location.

- ***Major participating components***

**Fig. 3:**
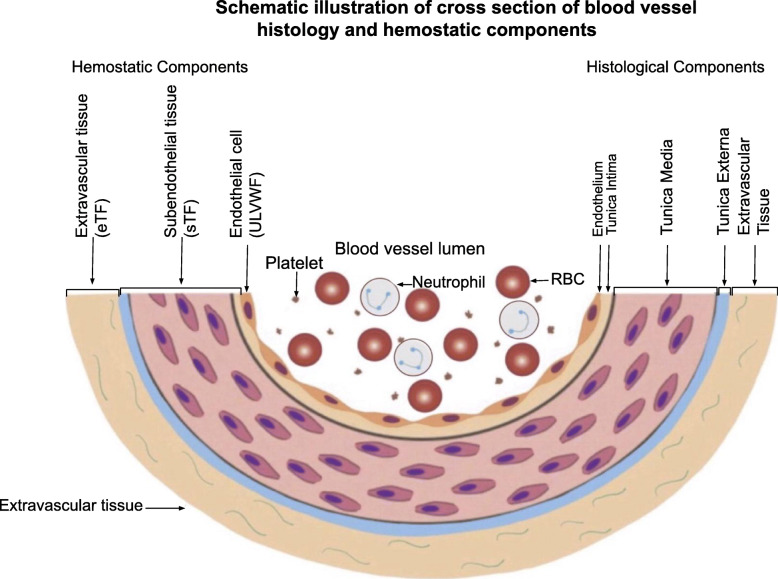
Schematic illustration of cross section of blood vessel and hemostatic components. (Reproduced and modified with permission from Chang JC. Clin Appl Thromb Hemost 2019 Jan-Dec; 25:1076029619887437). The most important part in the understanding of hemostasis in vivo is cognizance of the histological structure of the blood vessel and its role of vascular physiology in vascular injury. The reason is thrombosis cannot occur without vascular injury as explained in Table 3 and shown in novel “two-path unifying theory” of hemostasis [3, 4]. In intravascular injury, the local damage of ECs causes localized exocytosis of ULVWF to activate ULVWF path and damage extending into SET causes localized release of sTF to activate TF path. Both paths together lead to macrothrombogenesis and produce macrothrombus (e.g., DVT; pulmonary embolism; acute ischemic stroke). Should intravascular trauma extend further into EVT with additional release of eTF and bleed beyond EVT as seen in thrombo-hemorrhagic stroke, it produces macrothrombosis and hemorrhage (i.e., thrombo-hemorrhagic syndrome). Abbreviations: EVT, extravascular tissue; eTF, extravascular tissue factor; SET, subendothelial tissue; sTF, subendothelial tissue factor; RBC, red blood cells; ULVWF, unusually large von Willebrand factor multimers

Five major components presented in Table 3-(2) take part in hemostasis in vivo. From the point view of vascular wall physiology, two initiating hemostatic components are ULVWF located within ECs, and TF located in SET/EVT- subendothelial TF (sTF) from SET within the tunica intima, media and externa of the vascular wall and extravascular TF (eTF) from EVT outside of vascular wall - as illustrated in Fig. 3. In intravascular injury causing disseminated ECs damage (e.g., sepsis), TF is not available in the endothelium and in circulation [61, 62]. Thus, only microthrombi strings that are composed of platelet-ULVWF complexes [43, 63–66] are formed in endotheliopathy-associated “DIC” through lone activation of ULVWF path (Fig. 1) [3]. This is an extremely important concept in the understanding of EA-VMTD, including DIT as well as “DIC”. This vascular hemostatic component promotes the endothelial microthrombogenesis, leading to EA-VMTD.

- ***Vascular injury and hemostatic phenotype***

The physiological contribution of vascular wall damage is explained in Table 3-(3) and illustrated in Fig. 3. The cessation of hemorrhage, organization of hematoma, and formation of thrombosis are triggered by hemostasis in external bodily injury, internal tissue injury, or intravascular injury respectively. Thrombogenesis can specifically be defined as a blood clot forming process within the lumen of the blood vessel initiated by the breakdown of the endothelium with/without SET/EVT damage occurring in trauma or vascular disease. The essential event initiating the thrombogenesis is “intravascular injury” and the critical element determining the phenotype of the thrombotic disorder is the “level (depth) of vascular wall damage” [3, 4]. The level of damage may be (1) limited to ECs, (2) extended from ECs to SET, (3) further extended from ECs penetrating into SET and EVT beyond the vessel, and (4) localized within the tissue/organ from obtuse external trauma [60, 67].

The first (ECs damage) activates ULVWF path to produce microthrombi; the second (ECs and SET damage) activates both ULVWF and sTF paths to produce macrothrombus within the intravascular space; the third (ECs and SET/EVT damage) activates both ULVWF path and TF (sTF/eTF) path to produce combined macrothrombus without or with hemorrhage/hematoma penetrating beyond the vessel wall and into EVT; and the fourth (EVT damage alone) activates only TF path due to major internal EVT injury and produces tissue hemorrhage/hematoma made of some fibrin clots and blood cells. In the fourth situation, minor and small endothelial damage does not cause intravascular bleeding or thrombus formation because of higher pressure gradient within the vascular lumen compared to EVT [60]. These four different levels of vascular wall damage produce very different phenotypes of blood clots, each presenting with specific clinical thrombotic disorders (e.g., “DIC” or transient ischemic attack, acute ischemic stroke, thrombo-hemorrhagic stroke and acute hemorrhagic stroke) [60].

The thrombosis phenotype of “DIC” in sepsis occurs due to disseminated endotheliopathy and is characterized by microthrombi strings [6]. Following original publication of “two-path unifying theory” of hemostasis [3, 4], the updated version of hemostatic framework based on vascular wall physiology has been incorporated and published in subsequent articles as shown in Fig. 2a and b [17, 60, 67].

Pathologic hemostatic mechanism can be activated by few clinical conditions without vascular injury, but still utilizes a hemostatic path, either ULVWF or TF. TTP (i.e., GA-VMTD; AA-VMTD) utilizes activated “aberrant” ULVWF path due to ADAMTS13 deficiency and produces disseminated microthrombosis (i.e., DIT), and APL with consumption coagulopathy (true DIC) utilizes “aberrant” TF path due to abnormally expressed TF from leukemic promyelocytes and produces disseminated fibrin clot disease. These pathophysiological mechanisms are elaborated in previous publications [3, 4].

### Philosophical, physiological and phenotypical approach in identifying one hemostasis but three thrombogeneses

The theory on the mechanism of in vivo hemostasis has been proposed after philosophical, physiological and phenotypical contemplation about life in nature [4]. In the lexicon of hemostasis, at present the conceptual meanings of coagulation and thrombogenesis have been ambiguously defined. How can coagulation in external bodily injury be the same process to thrombogenesis in intravascular injury? The answer to this seminal question is very pertinent in the understanding of “how an underlying disease such as von Willebrand disease, hemophilia A, thrombocytopenia, thrombophilia and other diseases influences coagulation and thrombogenesis”. The fact is “coagulation” occurring in external bodily injury to stop the bleeding is one process. Still, it is the same process to “thrombogenesis” occurring in intravascular injury to produce to thrombosis. However, bleeding control is one event, but thrombogenesis contains three different sub-processes, which should be understood in the context of three different levels of vascular wall damage and activation of ULVWF and/or TF paths. Limited damage to ECs causes microthrombogenesis to generate microthrombi (e.g., “DIC”; transient ischemic attack [TIA]), limited activation of TF path causes fibrinogenesis to form fibrin clots (e.g., APL with consumption coagulopathy; tissue hematoma [e.g., subdural hematoma]), and combined damage to ECs and SET/EVT causes macrothrombogenesis to form macrothombus (e.g., DVT; acute ischemic stroke) [60].

The conception how could coagulation in bleeding of external bodily injury be one process of hemostasis in vivo, but thrombogenesis creates three different characters of blood clots in intravascular injury, is shown and summarized in Fig. 2b and Table 4. This very important question has been addressed by the physiological mechanism and phenotypical expression when the role of vascular wall damage was introduced in the framework of in vivo hemostasis. Please consider that contemporary in vitro hemostatic theory (i.e., extrinsic cascade) has been borne out from clotting studies in laboratory “test tubes”, in which the glass wall does not release ULVWF and TF, nor recruit the platelets from circulation.

**Table 4:** Classification of thrombotic disorder based on genesis of intrinsic characters of “blood clots”

	Microthrombosis	Fibrin clot disease	Macrothrombosis
**Level of vascular injury**	ECs (Level 1)	EVT (Level 2 only)	ECs/SET/EVT (Combined Level 3)
Activated path	ULVWF or aberrant ULVWF path	TF or aberrant TF path	Combined ULVWF & TF path
Mechanism	Microthrombogenesis	Fibrinogenesis	Macrothrombogenesis
Participants	ULVWF Platelet	TF Coagulation factors	ULVWF-platelet complexes Fibrin meshes NETs
**Character of thrombi**	Microthrombi	Fibrin clots	Macrothrombus
**Examples of disease**	VMTD Generalized: EA-VMTD (e.g., TTP-like syndrome, including false DIC) AA-VMTD* GA-VMTD* Local: Kasabach-Merritt syndrome Focal: TIA; HERNS syndrome	Hemorrhagic stroke: SDH Hemarthrosis APL-associated coagulopathy*	DVT PE Myocardial infarction Acute ischemic stroke Gangrene

*Abbreviations*: *APL* Acute promyelocytic leukemia, *DIC* Disseminated intravascular coagulation, “*DIC*”, False DIC, *DVT* Deep vein thrombosis, *HERNS* Hereditary endotheliopathy, retinopathy, nephropathy and stroke syndromes, *MI* Myocardial infarction, *NETs* Neutrophil extracellular traps, *PE* Pulmonary embolism, *SDH* Subdural hematoma, *TIA* Transient ischemic attack, *TF* Tissue factor, *ULVWF* Unusually large von Willebrand factor multimers, *VMTD* Vascular microthrombotic disease, *AA-VMTD* Antibody-associated VMTD, *EA-VMTD* Endotheliopathy-associated VMTD. GA-VMTD, gene mutation-associated VMTD. AA-VMTD*, GM-VMTD* and true DIC* in APL do not occur due to vascular injury, but utilizes hemostatic paths: aberrant ULVWF path or aberrant TF path (please see the text)

Armed with this fundamental derived from vascular wall physiology, the concept of in vivo hemostasis is reconstructed to explicate how hemostasis is initiated and leads to blood coagulation to make bleeding stop in external bodily injury, and the mechanism of thrombogenesis is proposed to explain how three different paths of blood clot formation take place in intravascular injury through one hemostatic system.

Certainly, both coagulation and thrombogenesis are mediated by the same physiological mechanism of normal hemostasis. Thus, the term “coagulation” in vivo and in vitro, “thrombosis” (microthrombosis and macrothrombosis) in vivo*,* “fibrin clot” in vivo and in vitro, and “blood clots” in vivo and in vitro which have been utilized in the terminology of hemostasis and thrombosis have to be reconciled within three mechanisms of thrombogenesis in one hemostatic system. While trying to reconstruct and connect dots in normal physiological mechanism, the role of NETosis in hemostasis [68] came into question, and certainly it couldn’t be ignored. To integrate its partaking in vivo hemostasis, an updated version of “two-path unifying theory” is proposed as shown in Fig. 2b. In intravascular injury, activated ULVWF path leads to microthrombogenesis and activated TF path leads to coagulation – more discrete term for “coagulation” is “fibrinogenesis” – and ULVWF path and TF path must participate together in macrothrombogenesis. As shown in Fig. 2a, activated ULVWF path from damaged ECs produces “microthrombi” strings and activated TF path from damaged SET/EVT produces “fibrin meshes” as represented in the schematic vascular wall structure in Fig. 3. Hence, depending upon the level (depths) of vascular injury, different phenotypes of the thrombotic disorder must occur in intravascular injury (i.e., ECs; ECs/SET; ECs/SET/EVT) as explained in earlier subheading of hemostasis “vascular injury in hemostasis phenotypes” [60].

### “Two-path unifying theory” of hemostasis

Philosophically and physiologically, it should be believed that nature must have endowed human with a simple, fast and efficient hemostatic mechanism to save lives in timely manner. Therefore, hemostasis must be a simple physiological mechanism evolved from biological adaptation and selection. In vascular wall injury, hemostasis should not be delayed by the process of complicated multiple bio-molecular steps following the injury. If there is any impediment, mediating through multiple activation of coagulation factors, and interactions of secondary coagulation components, cellular traps and molecular participation such as DNA and histones, this complexity of hemostasis is not compatible with human survival.

Instead, hemostasis should be an instantaneous and simultaneous process of activated ULVWF path and TF path starting at the gate of injured ECs and SET/EVT. We can observe these characteristics of blood clotting in external bodily injury site, bleeding time test in vivo and coagulation tests in vitro where no cellular traps and molecules are needed. Thus, this author would refute the concept of sequential hemostatic theory, theory of primary role of NETosis, and hypothesis of non-vascular organ system control as the main mechanism of hemostasis. Please note that the enthusiasm of NETosis has been cautioned and unconfirmed gaps remain in the interpretation among concerned molecular biologists [36, 37, 40, 41, 68].

Once the concept of ULVWF path is integrated into hemostasis to complement currently known one-sided TF path (activated TF-FVIIa cascade), normal in vivo hemostatic mechanism can be logically and systematically understood. According to “two-path unifying theory”, simultaneous activation of ULVWF path and TF path initiates hemostasis as shown in Fig. 2a. Two different clinical settings of vascular injury (i.e., external bodily injury and intravascular injury) require the participation of all five hemostatic components shown in Table 3-(2) to produce healthy hemostatic “blood clots” through the activation of cooperating ULVWF and TF paths.

In disseminated ECs damage from intravascular injury (e.g., sepsis-induced endotheliopathy leading to sepsis-associated coagulopathy), only ULVWF path is activated through platelet activation and endothelial exocytosis of ULVWF from Weibel-Palade bodies. The injury leads to excessive release of these prothrombotic multimers [43, 69–71]. ULVWF become anchored to the membrane of damaged endothelial cells (ECs) as long elongated strings with support of collagen [72] and recruit activated platelets. Both components together form platelet-ULVWF complexes, which become “microthrombi” strings. This process forming microthrombi strings in intravascular space is called microthrombogenesis [2–8]. The mechanism of ULVWF path in endotheliopathy mediating the expanded molecular response is presented separately in Fig. 1.

On the other hand, a local intravascular traumatic injury causing from ECs to SET/EVT damage (e.g., DVT), which results in additional local expression of TF, activates the TF/FVIIa complex-initiated fibrinogenetic path (i.e., TF path) as well as microthrombotic path (i.e., ULVWF path) at the local injury site. In contemporary coagulation theory, TF path, which fits properly with either the extrinsic coagulation cascade or cell-based coagulation [73], has been well formulated. To date, the fibrinogentic path in which TF-FVIIa-initiated coagulation cascade leads to the formation of fibrin clots [3] is considered to be the main body of hemostasis promoting every coagulation/thrombosis in vascular injury. Now, it becomes apparent that a newly incorporated ULVWF path shown in “two-path unifying theory” plays an equally or more important role in hemostasis in vivo [4, 17, 60, 67].

For example, “DIC” occurring in sepsis-initiated endotheliopathy is a partially activated hemostatic disease due to lone activation of ULVWF path, with serious pathophysiological ramification, resulting in DIT. Disseminated intravascular thrombosis is a form of EA-VMTD that may lead to hematologic phenotype TTP-like syndrome [17, 74].

## Endotheliopathy-associated vascular microthrombotic disease as new identity of “disseminated intravascular coagulation”

In Table 4, 3 different thrombotic diseases, which include 1) microthrombosis, 2) fibrin clot disease, and 3) macrothrombosis, can be clearly distinguished based on in vivo hemostasis model. This author’s point is that currently “DIC” is conceptualized as “fibrin clot disease” by most of authors although the pathology is clearly “microthrombosis”.

In Table 5-1, *Current concept-*based diagnostic criteria of “acute DIC” is defined as follows:

- It is a fibrin clot disease due to activated TF path.
- it is consumption coagulopathy.
- Consumption coagulopathy should result in decreased fibrinogen, decreased FVIII and decreased FV, and normal FVII.

**Table 5:** Diagnostic Criteria Comparison between acute DIC and EA-VMTD-associated HC

**1 Diagnostic criteria of “acute DIC” (conceptual)** ● Underlying disease - Sepsis and other critical illnesses (including COVID-19) ● Character of blood clots - Fibrin clots or microthromobi (different claims in the literature) ● Pathogenetic mechanism: consumption coagulopathy via TF path (resulting in consumption of platelets, fibrinogen, FVIII and FV.) ● Coagulation test results - Thrombocytopenia - Decreased fibrinogen, FVIII, FV (due to consumption) - Normal FVII (not consumed) - Prolonged PT - Prolonged aPTT - Positive D-dimer/FDP (due to fibrinolysis) ● Hematologic manifestations - Thromobocytopenia - MAHA **2 Diagnostic criteria of EA-VMTD-associated hepatic coagulopathy** ● Underlying disease - Sepsis and other critical illnesses (including COVID-19) ● Character of blood clots - Always microthrombi ● Pathogenetic mechanism: EA-VMTD via ULVWF path and hepatic involvement (resulting in consumption of platelets, endothelial exocytosis of ULVWF/FVIII, and decreased liver dependent factors.) ● Coagulation test results - Thrombocytopenia - Increased fibrinogen in early stage and decreased fibrinogen in late stage of liver disease (from liver necrosis in early stage and decreased synthesis in late stage) - Markedly increased ULVWF/VWF/VWF expression (due to exocytosis) - Markedly increased FVIII activity (due to exocytosis) - Markedly decreased FVII activity (due to liver damage) - Mildly decreased FV (due to liver damage) - Prolonged PT - Prolonged aPTT - Positive D-dimer/FDP (due to live tissue damage and fibrinogenolysis) ● Hematologic manifestations - Thrombocytopenia - MAHA	

*Abbreviation***:** *ARDS* Acute respiratory distress syndrome, **“***DIC*” False disseminated intravascular coagulation, *DIT* Disseminated intravascular microthrombosis, *HC* Hepatic coagulopathy, *MAHA* Microangiopathic hemolytic anemia, *MODS* Multiorgan dysfunction syndrome, *aPTT* Activated partial thromboplastin time, *PT* Prothrombin time, *SIRS* Systemic inflammatory response syndrome, *VWF* Von Willebrand factor multimers, *ULVWF* Unusually large von Willebrand factor multimers, *EA-VMTD* Endotheliopathy-associated vascular microthrombotic disease

However, the cases with “DIC” always presented with markedly increased FVIII [45, 75–77], increased expression of ULVWF/VWF [75–77] and increased fibrinogen in early stage and decreased in late stage of coagulopathy [45, 75], and decreased FVII [78]. Since these contradictions were so prominent in previously reported DIC articles, including recent cases of COVID-19 now, “DIC” diagnosis was not based on the coagulation factor assay but the scoring system. At this time, because COVID-19 coagulopathy is characterized by these unexpected coagulation factor test results, some coagulation specialists have called COVID-19-associated coagulopathy as DIC-like syndrome, hypercoagulability or dysregulated coagulation disease without proper explanation [75, 77, 79].

On the other hand, in Table 5-2, the diagnostic criteria of DIT (EA-VMTD)-associated coagulopathy is defined as follows:

- It is a microthrombotic disease due to activated ULVWF path.
- It is endotheliopathy-induced microthrombopathy causing liver necrosis and secondary hepatic coagulopathy.
- It should result in markedly increased activity of FVIII and overexpression of ULVWF/VWF from endothelial exocytosis, increased fibrinogen in early stage (due to release from hepatocytes) and decreased fibrinogen in late stage (due to impaired live function), moderate reduction of FVII.

Indeed, previous publications with the diagnosis of DIC [45] and recent publications on COVID-19-associated coagulopathy unequivocally [75–77] confirm: 1) DIT-associated hepatic coagulopathy is exactly the same to current concept of “DIC” in coagulation studies on “real” patients, 2) DIT (EA-VMTD)-associated with hepatic coagulopathy is perfectly congruous with current concept of acute “DIC” in every coagulation study presenting on “real” patients, as well as DIC-like syndrome, poorly defined hypercoagulability, and dysregulated hemostatic disease. Finally, the term EA-VMTD resolves the ambiguity of microthrombosis amongst “DIC”, TTP and TTP-like syndrome, hemolytic uremic syndrome and thrombotic microangiopathy. In conclusion, acute DIC does not exist. EA-VMTD with or without hepatic coagulopathy explains every poorly defined coagulopathy in sepsis and other critical illnesses.

When a patient presents with VMTD in sepsis or critical illness with clinical features characterized by microthrombosis, thrombocytopenia, inflammation, and MODS such as altered mental state and acute renal failure, clinicians typically include “DIC”, TTP, TTP-like syndrome, diffuse encephalopathic stroke, HUS, and thrombotic microangiopathy in the differential diagnosis. Now, TTP-like syndrome is found to be the best term to be used all of the above conditions except TTP. It is very common condition and can be defined as “EA-VMTD” because TTP-like syndrome is always associated with endotheliopathy [6]. In contrast, TTP occurs due to ADAMTS13 deficiency that is associated with hereditary disease (GA-VMTD) or associated with acquired autoimmune disease (AA-VMTD). HUS is a descriptive term for only representing one organ (kidneys) phenotype and diffuse encephalopathic stroke is that of another organ (brain) phenotype among MODS caused by EA-VMTD [17]. Additionally, thrombotic microangiopathy does not represent a disease, but only provides a pathophysiologic descriptive term if present with thrombocytopenia and MAHA in EA-VMTD.

In retrospect, “DIC” was not the best term to denote as “disease” because “coagulation” does not represent “disease”, but it evokes the connotation of “clotting process” without a clear meaning of pathology such as thrombosis or clinical phenotype such as stroke or heart attack. As mentioned earlier, it could have been better if the term disseminated intravascular (micro) thrombosis (DIT) has been used instead of “DIC” because in the lexicon of hemostasis, every blood clot has been designated with the term “thrombosis” if the platelet is participated.

There is another predicament with the diagnosis of “DIC” and true DIC. Two pathogenetically different conditions have been called DIC; one is “DIC” of sepsis and other critical illnesses, and the other is true DIC of APL-associated hemorrhagic disease. The “DIC” of sepsis is clearly EA-VMTD that is due to microthrombi composed of platelet-ULVWF strings following activation of ULVWF path, but the DIC of APL is literally “disseminated fibrin clot disease” that occurs due to fibrin clots formation following activation of TF path as shown in Tables 1 and 4. Thus, DIC of APL shouldn’t be included in VMTD, but it should remain as true DIC or “disseminated fibrin clot disease”.

Within the term disseminated VMTD, there are three pathologic entities present: 1) GA-VMTD (i.e., hereditary gene mutation-associated TTP), 2) AA-VMTD (i.e., acquired autoimmune TTP), 3) EA-VMTD (i.e., endotheliopathy-associated TTP-like syndrome). After exclusion of GA-VMTD and AA-VMTD, all other disseminated microvascular diseases with or without the triad of thrombocytopenia, MAHA and one or more organ dysfunction syndrome, including “DIC”, HUS, and thrombotic microangiopathy, should be classified as EA-VMTD (i.e., TTP-like syndrome if the triad is present), which molecular pathogenesis and characteristics of clinical and hematologic syndromes are exactly the same and concisely explained [6] and reiterated in Table 1, Tables 2 and 4 for clarity of the identical nature of “DIC” and EA-VMTD.

From this complexity, a simplified collective concept of EA-VMTD has emerged. The diversity of organ localization should be understood from endothelial heterogeneity of the host and organotropism of the pathogen, which create different unique organ dysfunction syndromes resulting in organ endotheliopathy [17]. When EA-VMTD is complicated by respiratory failure due to pulmonary vascular involvement of microthrombosis, leading to pulmonary hypoxia, it is called “acute respiratory distress syndrome (ARDS)”. Likewise, when EA-VMTD is complicated by hepatic coagulopathy due to liver damage from microthrombosis, leading to liver necrosis, it is called acute liver failure or fulminant hepatic failure instead of previously termed acute “DIC”. Veritably ARDS, acute liver failure, and fulminant hepatic failure as well as hemolytic-uremic syndrome are well known to be associated with TTP-like syndrome.

### Mechanisms involved in microthrombogenesis

#### Complement activation and endotheliopathy

In addition to sepsis, complement activation is well documented in many non-septic critical illnesses such as head trauma and brain injury [80, 81], autoimmune diseases [82], complications of pregnancy (e.g., preeclampsia, amniotic fluid embolism) [83], cancer [84, 85], and transplant [86]. The complement system provides vital defense against invading pathogens and certain toxins. This activation is principally mediated through the deposition of C3b to pathogenic surfaces and host endothelium. If the deposited C3b is not appropriately regulated, there is progression to terminal pathway of complement activation via the C5 convertases, generating the potent anaphylatoxin C5a and C5b-9 (membrane attack complex [MAC]). Unsurprisingly, MAC has the potential to cause injury to innocent bystander host endothelium [87].

The patients with TTP-like syndrome, including “DIC”, are associated with complement activation and characterized by thrombocytopenia, MAHA, and microvascular thrombosis [86–90], which can be explained by endothelial molecular pathogenesis as illustrated in Fig. 1. Certainly, unnecessary activation of complement system could become detrimental to the host. Additionally, “DIC” and TTP-like syndrome often present with MODS. If we were to separate “DIC” and TTP-like syndrome as two different diseases and accept the notion that two conditions only share the same pathological mechanism [16], how can we explain their identical hematologic features with the same microvascular thrombosis, complement activation and endotheliopathy? In every respect, “DIC” is identical to TTP-like syndrome caused by EA-VMTD [6].

#### Endotheliopathy and hemostasis

When the C5b-9 complex formed from complement activation provokes endothelial injury, the endotheliopathy is suspected to trigger structural and biological changes of ECs and initiates molecular dysfunction. Endotheliopathy simultaneously activates two molecular pathways: inflammatory and microthrombotic. The activated inflammatory pathway triggers inflammation, and activated microthrombotic pathway promotes microthrombogenesis. The molecular response to activated inflammatory pathway is the release of various proinflammatory cytokines such as interleukin-1, interleukin-6, tumor necrosis factor-α, interferon-γ and many others [91, 92]. But the molecular response to activated microthrombotic pathway is endothelial exocytosis of ULVWF from Weibel-Palade bodies [93] and platelet activation [94] that mediate microthrombogenesis [2–7]. This microthrombogenesis produces the platelet-ULVWF complexes, which strings are anchored to the damaged ECs and lead to microthrombosis, obstructing the microvasculatures. In sepsis, EA-VMTD initially presents with the clinical picture of chronic “DIC”. Later on in certain patients, due to endothelial heterogeneity of the host and/or organotropism of the pathogen, endotheliopathy may preferentially involve in the liver resulting in liver necrosis as a phenotype of MODS and leads to hepatic dysfunction. This results in hepatic coagulopathy, which is currently being termed wrongfully as acute “DIC”.

#### Hemostasis and vascular microthrombotic disease

Furthermore, the intrinsic character of the platelet-ULVWF complex in every TTP-like syndrome should be identical, which becomes microthrombi strings. All the VMTD occur as a result of microthrombogenesis although microthrombi in TTP are formed in microcirculation [14, 66] as a result of aberrant activation of ULVWF path without vascular injury, but microthrombi of TTP-like syndrome, including “DIC”, are produced at the injured endothelial cells [2–7, 69] as a result of lone activation of ULVWF path. Red cell fragmentation is suspected to occur when red blood cells pass through between microthrombi strings anchored to the ECs within the capillaries and arterioles.

### Differential diagnostic tests between acute “DIC” and EA-VMTD associated hepatic coagulopathy

Finally, how to differentiate 1) acute “DIC” (theoretical), 2) EA-VMTD-associated “hepatic coagulopathy” and 3) coagulopathy of APL? In hematologic evaluation of critically ill patients, the contemporary diagnosis of acute “DIC” has been established whenever an unexplained coagulopathy presents with laboratory features of thrombocytopenia, prolonged activated thromboplastin time and prothrombin time, positive D-dimers and decreasing fibrinogen level. However, this combination feature is not diagnostic of theoretical DIC.

To make accurate differential diagnosis of coagulopathy between acute DIC (theoretical) and EA-VMTD-associated hepatic coagulopathy, more specific laboratory tests are needed with proper interpretation based on pathogenetic mechanisms, which are listed in Tables 2 and 5 with following characteristic features.

- FVIII activity (must be decreased in acute DIC, but markedly increased in EA-VMTD-associated hepatic coagulopathy)
- FV activity (must be decreased in acute DIC, and decreased in EA-VMTD-associated hepatic coagulopathy)
- FVII activity (must be normal in acute DIC, but moderate to severely decreased in EA-VMTD-associated hepatic coagulopathy)
- Fibrinogen level (must be markedly decreased in DIC, but markedly increased in early stage of EA-VMTD-associated hepatic coagulopathy and markedly decreased in late stage)
- VWF/ULVWF expression (not applicable in acute DIC, but markedly increased in EA-VMTD-associated hepatic coagulopathy)

In theoretical acute DIC, decreased FVIII and FV activity must be present due to inactivation of FVIIIa and FVa via activated protein C pathway and protein S pathway if TF-FVIIa coagulation cascade had been involved. However, the rest of coagulation factors FIIa, FVIIa, FIXa, and FXa should be normal because they are not inactivated [95, 96]. In hepatic coagulopathy, FII, FV, FVII, FIX, and FX may be mild to moderately diminished. In endotheliopathy, EA-VMTD is characterized by markedly increased activity of FVIII and overexpression of VWF/ULVWF due to their exocytosis from Weibel-Palade bodies.

An extremely interesting finding in acute hepatitis patients with severe hepatic coagulopathy, acute liver failure and fulminant hepatic failure has shown markedly increased FVIII activity and increased expression of VWF [46, 97–104]. This unique finding of FVIII has not been explained to date. Some blamed it to “non-specific but acute phase reactant” in acute illnesses and insisted it is a contributing factor to hypercoagulable state. This author affirms both increased activity of FVIII and overexpressed VWF/ULVWF are the specific biomarkers for activated ULVWF path associated with endotheliopathy [105] occurring in EA-VMTD. Therefore, increased FVIII and VWF activity in critical illnesses such as sepsis (e.g., COVID-19) [75–77] and trauma should alert the potential of underlying, but masked, EA-VMTD. This increased activity of FVIII is not the cause of hypercoagulable state, but is the result of on-going covert microthrombogenesis, leading to EA-VMTD, in critically ill patients. No doubt, sepsis-associated coagulopathy is not “DIC”, but is endotheliopathy-associated vascular microthrombotic disease.

## Conclusion

Sepsis-associated coagulopathy (“DIC”) has been conceptualized as a coagulation disorder occurring as a result of activation of TF path from TF-FVIIa coagulation cascade. Now, based on coagulation data analysis and two novel hemostatic theories, sepsis-associated coagulopathy is identified to be a microthrombotic disorder occurring as a result of lone activation of ULVWF path and endotheliopathy-initiated microthrombogenesis. EA-VMTD without coagulopathy has been designated to be “chronic”, “compensated”, or “covert” DIC, and EA-VMTD with hepatic coagulopathy due to microthrombosis has been designated to be “acute”, “uncompensated”, or “overt” DIC. Now, it is time to delete the term “DIC” and should be replaced by correct term EA-VMTD, which also explains why “DIC” is often manifested with TTP-like syndrome and coexisted with hepatic coagulopathy. The therapeutic approaches may include recombinant ADAMTS13 or N-acetylcysteine to directly target microthrombogenesis, or anticomplement agent or therapeutic plasma exchange to indirectly inhibit endothelial molecular pathogenesis [5, 17, 67, 106, 107].

## Data Availability

“Data sharing not applicable to this article as no datasets were generated or analyzed during the current study. If you do not wish to publicly share your data, please write: “please contact author for data requests.”

## Abbreviations

APC: Activated protein C
APL: Acute promyelocytic leukemia
ARDS: Acute respiratory distress syndrome
DIC: Disseminated intravascular coagulation
“DIC”: false disseminated intravascular coagulation
DIT: Intravascular microthrombosis
ECs: Endothelial cells
DVT: Deep vein thrombosis
EVT: Extravascular tissue
FVIIa: Activated factor VII
HUS: Hemolytic-uremic syndrome
MAC: Membrane attack complex
MAHA: Microangiopathic hemolytic anemia
MODS: Multi-organ dysfunction syndrome
NET: Neutrophil extracellular traps
SET: Subendothelial tissue
TF: Tissue factor
TIA: Transient ischemic attack
TTP: Thrombotic thrombocytopenic purpura
VWF: Von Willebrand factor
ULVWF: Unusually large von Willebrand factor
VMTD: Vascular microthrombotic disease
AA-VMTD: Antibody-associated VMTD
EA-VMTD: Endotheliopathy-associated VMTD
GA-VMTD: Gene mutation-associated VMTD
